# Genetic and genomic analysis of Belgian Blue’s susceptibility for psoroptic mange

**DOI:** 10.1186/s12711-024-00921-7

**Published:** 2024-07-05

**Authors:** Roel Meyermans, Steven Janssens, Annelies Coussé, Susanne Tinel, Wim Gorssen, Fabrice Lepot, Xavier Hubin, Patrick Mayeres, Wim Veulemans, Nathalie De Wilde, Tom Druet, Michel Georges, Carole Charlier, Edwin Claerebout, Nadine Buys

**Affiliations:** 1 Center for Animal Breeding and Genetics, Department of Biosystems, KU Leuven, 3001 Leuven, Belgium; 2 Elevéo Asbl, 5590 Ciney, Belgium; 3 CRV, 6843 NW Arnhem, The Netherlands; 4 Department of Translational Physiology, Infectiology and Public Health, UGent, 9820 Merelbeke, Belgium; 5 Unit of Animal Genomics, GIGA Institute and Faculty of Veterinary Medicine, Liège University, 4000 Liège, Belgium

## Abstract

**Background:**

Psoroptic mange, caused by *Psoroptes ovis* mites, is affecting Belgian Blue cattle’s welfare and production potential. The Belgian Blue cattle—known for its high degree of muscling, low feed conversion ratio and high beef quality—is highly susceptible for this disease.

**Results:**

In this study, we phenotyped 1975 Belgian Blue cattle from more than 100 different groups on commercial beef farms for their psoroptic mange susceptibility. Substantial individual differences were observed within these management groups, with lesion extent differences up to ± 15%. Animal models showed that estimated heritabilities were low for lesion extent and severe lesion extent (0.07 and 0.09, respectively) and 0.12 for the number of mites. A genome wide association study for mange susceptibility revealed signals on BTA6, BTA11, BTA15 and BTA24. In these regions, candidate genes *GBA3*, *RAG2*, and *TRAF6* were identified.

**Conclusions:**

Despite the challenges in phenotyping for psoroptic mange due to the timing of screening, the continuous evolution of lesions and different management conditions, we successfully conducted a study on the genetic susceptibility to psoroptic mange in Belgian Blue cattle. Our results clearly indicate that psoroptic mange is under polygenic control and the underlying candidate genes should be studied more thoroughly. This is the first study providing candidate genes for this complex disease. These results are already valuable for Belgian Blue breeding, however, further research is needed to unravel the architecture of this disease and to identify causal mutations.

**Supplementary Information:**

The online version contains supplementary material available at 10.1186/s12711-024-00921-7.

## Background

Belgian Blue cattle has been selected primarily for high muscularity, resulting in a high efficiency for beef production, both for lean meat and cutting out percentages. However, the Belgian Blue breed is also confronted with a skin disease, called psoroptic mange [1, 2]. This disease, caused by the ectoparasitic mite *Psoroptes ovis*, is characterized by severe dermatitis and pruritus [3–5]. Psoroptic mange results in thick crusty skin lesions that are prone to secondary bacterial infections. Moreover, due to the intense pruritus, animals start licking, biting and scratching their wounds, resulting in self-trauma. Subsequently, this leads to hair loss, more skin damage, bleeding and even more bacterial infections. In severe cases, infestations lead to economic losses due to factors such as weight loss, reduced feed efficiency, additional veterinary treatment costs and reduced leather quality at slaughter, or even death in case of young animals [2, 5]. This disease clearly impairs animal welfare as shown by scratching, restlessness and reduced foraging and ruminating behavior of affected animals.

The Belgian Blue breed is more susceptible to psoroptic mange than other breeds. Their (genetic) predisposition was first described by Pouplard and colleagues [1], and was later confirmed by Losson and colleagues [2]. Sarre et al*.* reported a psoroptic mange incidence of 74% in Belgian Blue farms in Flanders (Northern Belgium) [6]. Moreover, they found that almost half of the Flemish cattle farmers had difficulties controlling the disease and a large number of them underestimated the psoroptic mange problem. Later, Sarre and colleagues compared infested Belgian Blue to infested Holstein and reported differences in the immune response, with increased levels of IL-17 in the skin and IFN-γ in re-stimulated immune cells in Belgian Blue cattle [7]. Chen et al*.* recently suggested that a stronger Th2-type response could underly the increased susceptibility [8].

Currently, farmers control the disease via acaricides. However, multiple studies have shown that disease control often fails [6, 9]. Moreover, acaricide resistance of *P. ovis* against the commonly used macrocyclic lactones has been observed on a large number of farms. Therefore, it is important to investigate alternatives to fight this disease. A promising way is to focus on the host genetics. Individual differences in sensitivity within Belgian Blue herds can frequently be observed and improving genetic resilience towards psoroptic mange may offer a sustainable solution.

Investigating the genetic predisposition of the host’s sensitivity to parasite infestations in cattle is not new. Indeed, it has already been confirmed by Mapholi and colleagues that several regions (quantitative trait loci, QTL) in the bovine genome were associated with tick resistance in Nguni cattle [10]. Here, tick resistance was quantified by the number of ticks that was counted monthly on each animal. The count of ticks showed a non-zero, but low heritability, ranging from 0.02 to 0.17. Moreover, Burrow found moderate to high heritabilities for tick and gastrointestinal nematode load (0.35 to 0.44) in Belmont Red cattle [11]. May et al*.* reported low heritabilities for gastrointestinal nematode fecal egg load (0.05) and moderate heritabilities for liver flukes (0.33) (*Fasciola hepatica*), and accounted the low heritability to the large environmental effect [12]. Twomey et al*.* also found a low (0.09) heritability for liver flukes in dairy cattle [13]. May and colleagues used a genomic approach to study infestations with endoparasites (gastrointestinal nematodes, liver flukes and bovine lungworms *Dictyocaulus viviparus*) in German Black Pied cattle, found 23 candidate genes linked to disease resistance (of which 5 were linked to immune response mechanisms), but concluded that the endoparasitic disease resistance was under polygenic control [14].

Previous research in a sub-population of Belgian Blue cattle showed an association between mange sensitivity and the *nt821*(*del11*) mutation in the *MSTN* gene responsible for the double muscling phenotype [15]. Here, animals homozygous for the *nt821*(*del11*) mutation had significantly larger lesions, compared to animals with the wild type allele. Therefore, one could expect higher levels of mange susceptibility in the main Belgian Blue population. However, as the majority of the Belgian Blue population is homozygous mutated for *MSTN*, this association cannot explain the observed differences in susceptibility within the breed [15].

As low to moderate heritabilities for both endo- and ectoparasites have been shown in the past, it encourages the exploration of Belgian Blue cattle’s sensitivity to *P. ovis* and to uncover the underlying genomic architecture of this sensitivity to these parasites. Therefore, we initiated a large genetic study for psoroptic mange sensitivity in Belgian Blue cattle using both quantitative and molecular approaches.

## Methods

### Animal sampling

A total of 1975 Belgian Blue cattle were phenotyped and sampled on commercial beef farms in two consecutive projects (Project 1: 2013–2015, n = 669, and Project 2: 2018–2019, n = 1306). We recorded the within farm management group (called contemporary groups, CG, n = 139) for each animal. These groups comprise animals that share similar management practices, including prior acaricide treatments (if applicable), summer grazing or feeding routines, and that often fall within the same age class. Only herds with clinical signs of mange were sampled to exclude false-negative results. Mange sensitivity was screened in both projects by recording the lesion extent (LE) (as % of body surface) following [16]. In Project 2, lesions were further grouped into four categories: from 1 (almost healed) to 4 (severe active infestation) as shown in Additional file 1 Fig. S1. Severe lesion extent (SLE) was defined as the total lesion extent of the most severe lesions (score 3 and 4) and was available for 1306 animals sampled in Project 2. Moreover, in Project 2, three skin scrapings were taken at the predilection sites for *P. ovis* (tail, back and neck) to assess the number of live *P. ovis* mites (mite count, MC; total surface area: 12 cm^2^) [up to 1000 mites in the sample (only 1 animal had more mites present in the sample)]. Likewise, the presence of other ectoparasites (e.g. *Chorioptes bovis*, *Bovicola bovis*, *Haematopinus eurysternus*) was recorded. In Project 1, only the presence of live *P. ovis* and other parasites was recorded per animal and unfortunately no quantitative count was performed. Pedigree data were provided by the respective Belgian Blue herdbooks (CRV, The Netherlands and elevéo/awé, Belgium). Unfortunately, not all sampled animals were registered in a herdbook (e.g. calves that were not considered as breeding animals) and age at sampling was therefore only available for 1504 animals. For 790 animals (Project 2), also skin thickness was measured using a Harpenden Skinfold Caliper at the height of the ribs and Pearson correlations with LE, SLE and MC were calculated using R (*cor* function).

CGs were only considered for sampling when the animals had not undergone acaricide treatments for a minimum of six weeks beforehand. This was to eliminate the possible influence of potential misapplication of acaricides (over- or underdosing of animals), as reported by [6], which would bias phenotyping. Moreover, as all animals were grouped by their CG, all animals were treated at the same moment, with similar dosing. The majority of the animals was sampled upon entering their winter stables (October–December) and was therefore not treated in the past six months (spring and summer), as most farmers treat their animals for the first time at the start of the winter. When allowed by the farmers, a second visit was performed approximately two weeks after the first sampling and acaricide treatment was postponed. During this second visit, MC was not recorded. In total, 665 records were obtained from animals sampled again after two weeks to assess the phenotyping repeatability.

### Genotyping

Genomic DNA was extracted either from ear notches or whole blood (EDTA). Genotyping was performed on the Illumina BovineSNP50 array v2 (54,607 SNPs) for animals sampled in Project 1 (n = 669), on the Illumina BovineSNP50 array v3 (53,218 SNPs) for 96 animals from Project 2, and on the EuroGenomics MD BeadChip array (52,397 SNPs) for 1133 animals sampled in Project 2 (Illumina, San Diego, CA, USA). Genotype quality control was performed using PLINK 1.9 [17] following Anderson et al*.* [18]. Animals with low call rate (< 95%) and outlying heterozygosity rate (> 3 SD) were discarded from the analysis. A sex check was performed to detect potential mis-identification of individuals [18]. Individual quality control resulted in 1701 animals retained for further analysis. At SNP level, the following quality controls were performed: (1) only autosomal SNPs and SNPs with known genomic location were retained, (2) only SNPs with high call rate (> 0.95) were kept, (3) SNPs with low minor allele frequency (< 0.01) were discarded, and (4) SNPs with significant deviation from the Hardy–Weinberg equilibrium (P-value < 0.0001) were also rejected. After quality control, the overlap between the three different genotyping arrays was 36,817 SNPs for 1701 genotyped Belgian Blue cattle. Linkage disequilibrium pruning was performed at a level of 0.5 R^2^ (discarding 7807 SNPs) retaining 29,010 SNPs.

### Genetic parameters for psoroptic mange

Heritabilities (h^2^) for LE, SLE and MC were estimated using a multiple trait animal model with single step GBLUP (ssGBLUP) using the blupf90 family programs (renumf90 and remlf90) [19]. SLE and MC were only evaluated for 1306 animals sampled in Project 2. To model the additive genetic effect, also including animals that were not successfully genotyped, pedigree information was extracted for all phenotyped individuals up to five generations ago (total n = 9091) and joined with the available genotypes to create an H-matrix (combined pedigree (A) and genomic (G) relationship matrix). Heritabilities were estimated as the proportion of additive genetic variance over the total phenotypic variance. A random CG effect was included to model LE, SLE and MC and was also expressed proportionally to the phenotypic variance (c_g_^2^). Animal models were of the form

$$\mathbf{y}=\mathbf{X}\mathbf{b}+\mathbf{Z}\mathbf{a}+\mathbf{W}\mathbf{c}+\mathbf{e}$$

where $\mathbf{y}$ is the vector of phenotypes, $\mathbf{b}$ the vector assigning the fixed effects (for LE and SLE: sex, coat color (white, blue and black), birthyear (n = 11), age at moment of phenotyping, their respective project (n = 2); and for MC: sex), $\mathbf{a}$ is the vector of additive genetic effects (n = 9091), $\mathbf{c}$ is the vector of CG effects (n = 109), and $\mathbf{e}$ is the vector of residual effects. $\mathbf{X}$, $\mathbf{Z}$ and $\mathbf{W}$ are the respective design matrices for fixed, additive genetic, and random CG effects, respectively. Minimum group size per CG was set to five, and groups with fewer observations (n = 29) were assigned to one CG group. The multiple-trait model was applied using animals with complete observations for all fixed effects (n = 1504 for LE, n = 1057 for SLE and MC), with the age at the time of sampling being the most limiting factor. Genetic correlations between skin thickness and LE, SLE and MC were computed in three bivariate models with the same fixed and random effects as indicated above using REMLF90. Sampling errors (SE) were assessed using AIREMLF90 following Houle & Meyer [20].

### Genome wide association study

GWAS analyses were based on two approaches: a case/control approach and a quantitative approach on LE, SLE and MC. Case and control selection was performed on extreme phenotypes within CGs, where ± 10% animals with the highest LE and SLE were classified as sensitive for psoroptic mange (case). The animals with the ± 10% lowest (S)LE per CG were identified as resilient for psoroptic mange (control).

To increase power, a haplotype-based approach was used instead of a single-SNP approach [21]. At medium SNP density, GWAS were performed on the set of 29,010 SNPs which were first phased using BEAGLE 3.3.2 [22]. The haplotypes were assigned to clusters of similar haplotypes, referred to as ancestral haplotypes, by a chromosome-by-chromosome hidden Markov model using HiddenPHASE 1.1 [23] with the number of clusters set to 20. To confirm the results at medium SNP density level, genotypes were imputed to a higher density level (633,512 SNPs) using BEAGLE 3.3.2 and a Belgian Blue reference population (elevéo/awé, Belgium). Clustering of haplotypes was performed using HiddenPHASE 1.1 with 10 ancestral haplotypes instead of 20, as at higher density (and shorter segment length) the haplotypes become more ancient and are fewer. Haplotype-based GWAS were conducted via a linear mixed-model approach using the GLASCOW software [21]. This method accounts for possible stratification that could be originating from hidden population or family structure and estimates the genomic relationship matrix using the ancestral haplotypes [21]. For the case/control approach, phenotypes were first converted using the logit link function. The mixed model included sex, project, birthyear, coat color and (random) CG effects. Suggestive signals were identified at p-value < ${10}^{-5}$ and genomic significance was set at a Bonferroni-corrected significance of 0.05 (${10}^{-5.9}$ for medium density data and ${10}^{-7.1}$ for high density data). GWAS results were visualized using the qqman package in R [24]. Genes in regions of interest were identified using the R package biomaRt [25, 26].

## Results

### Descriptive statistics

**Fig. 1:**
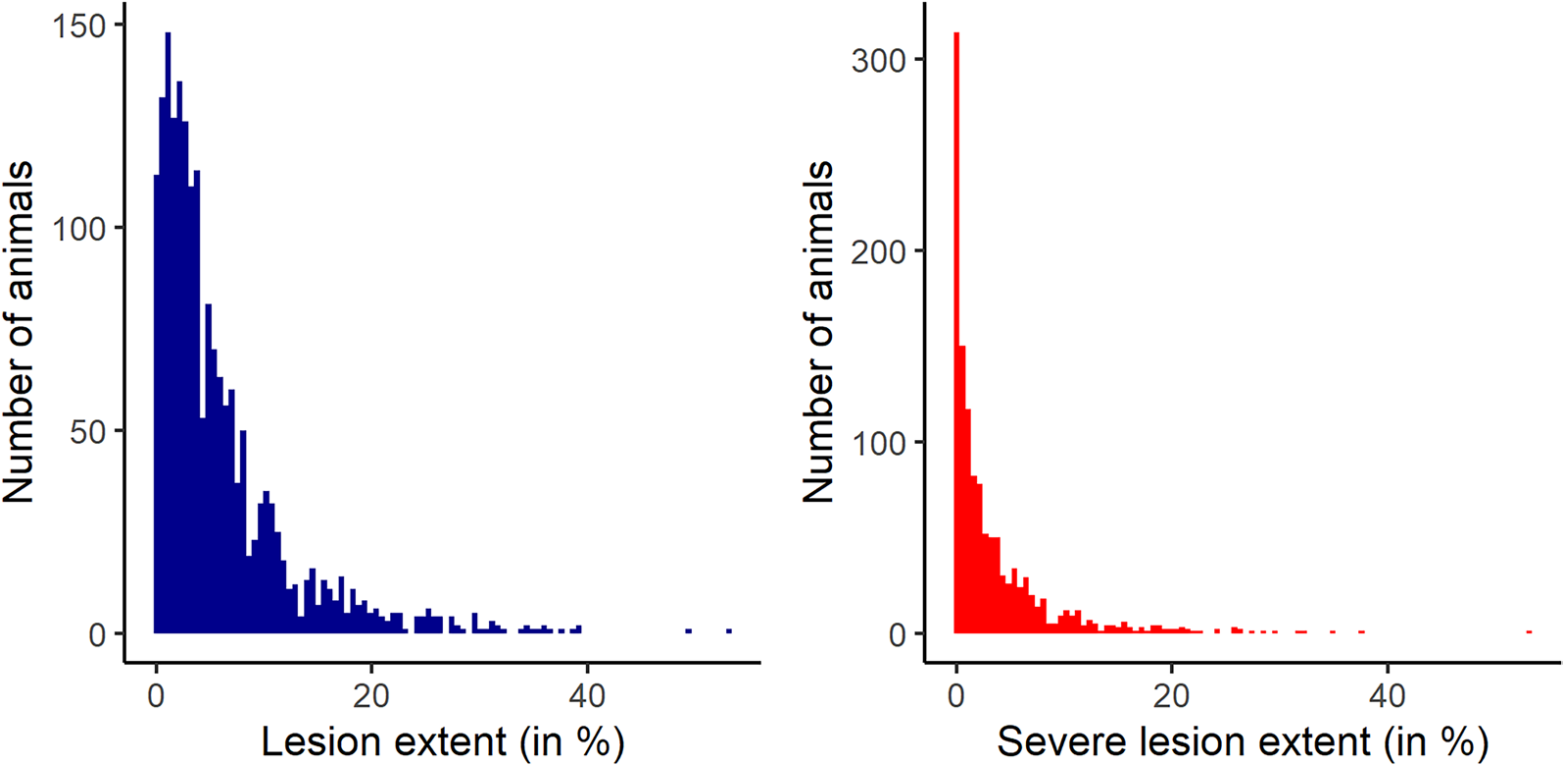
Histograms of lesion extent (LE) and severe lesion extent (SLE). LE and SLE are expressed as % of body surface of the animal and show a right-skewed distribution of psoroptic lesion extent

Figure 1 shows the distribution of LE and SLE for all animals and detailed descriptive statistics for LE, SLE, MC and skinfold thickness are shown in Table 1. Pearson correlations were 0.81 between LE and SLE, 0.25 between LE and MC, and 0.26 between SLE and MC. Phenotype distributions were right skewed: 112 animals had 0% LE, 311 animals had 0% SLE and 1095 animals had a mite count of zero. 93% of all examined animals were cows, 7% were bulls. 30% of the sampled cattle had a white coat color, 54% a blue coat, and 16% a black and white coat. Average age of the sampled animals was 34 months (SD: 20.3 months). Skinfold thickness measurements were found to be not correlated with both LE, SLE and MC (r = − 0.03, − 0.04 and − 0.07, respectively). The average LE within CGs ranged 14.67% (9.82% for SLE) and the average standard deviations of LE and SLE within CGs were 4.13% and 3.01%, respectively. Detailed lesion statistics and histograms per appearance score are given in Additional file 2 Table S1 and Additional file 3 Fig. S2. A histogram of all mite counts is shown in Additional file 4 Fig. S3. 655 animals were sampled a second time after two weeks. Correlations for LE and SLE between successive samplings were 0.74 and 0.71, respectively.

**Table 1:** Descriptive statistics of psoroptic mange lesions, mite counts and skin thickness for the sampled Belgian Blue cattle

	Abbreviation	N	Mean	Median	Range	SD
Lesion extent (in %)	LE	1975	5.85	3.72	0–53.14	6.44
Severe lesion extent (in %)	SLE	1306	3.38	1.43	0–53.14	5.15
Mite count (on 12 cm^2^)	MC	1306	21.03	0	0–1000	73.23
Skin thickness (in mm)		790	9.19	9.20	4.0–15.2	1.69

N: Number of available phenotypes for each phenotype

### Genetic parameters for psoroptic mange

Estimates of the multiple trait model for the variance components, h^2^ and c_g_^2^, are shown in Table 2. In general, heritabilities for LE, SLE and MC were low (0.07–0.12), but c_g_^2^ estimates were considerably higher (0.15–0.42), indicating a large proportion of the observed variance can be attributed to the different CGs. When estimating heritabilities with single-trait models using the same random and fixed effects, similar h^2^ and c_g_^2^ were found (0.06 and 0.44 for LE, 0.08 and 0.56 for SLE, and 0.11 and 0.17 for MC). Genetic correlations between LE and SLE were estimated to be 0.40 (SE = 0.01), between LE and MC to be 0.04 (SE = 0.01), and between SLE and MC to be 0.09 (SE = 0.01). The estimated correlation for the CG effect between LE and SLE was 0.90 (SE = 0.02), between LE and MC was 0.38 (SE = 0.05), and between SLE and MC was 0.40 (SE = 0.04). Estimates of the fixed effects of the animal model are shown in Table 3. Genetic correlations between skinfold thickness and LE, SLE and MC, estimated with three bivariate models, were 0.04 (SE = 0.93), − 0.10 (SE = 0.66) and − 0.02 (SE = 0.18), respectively.

**Table 2:** Estimated genetic parameters for psoroptic mange lesions and mite counts in Belgian Blue

Trait	Additive genetic variance	CG variance	Residual variance	h^2^	c_g_^2^	N
LE	3.06 (1.30)	19.06 (3.04)	23.27 (1.45)	0.067 (0.03)	0.420 (0.04)	1504
SLE	3.17 (1.00)	17.12 (3.41)	14.40 (1.16)	0.091 (0.03)	0.494 (0.05)	1057
MC	516.20 (180.41)	622.50 (130.38)	3042 (190.25)	0.124 (0.04)	0.149 (0.03)	1057

*LE* lesion extent, *SLE* severe lesion extent, *MC* mite count, *CG* contemporary group, *h*^*2*^ heritability, *c*_*g*_^*2*^ proportion of variance explained by contemporary group effects. N is the number of animals phenotyped for the trait. The standard error (SE) of the estimates is given between brackets

**Table 3:** Fixed effects estimates for lesion extent (LE), severe lesion extent (SLE) and mite count (MC

	N	LE	SLE	MC
Sex				
Female	1397/1013	4.41 (1.71)	3.18 (0.88)	12.11 (7.56)
Male	107/44	7.67 (1.80)	5.43 (1.81)	34.37 (9.67)
Coat color				
White	454/297	1.22 (0.45)	0.73 (0.39)	–
Blue	813/565	0.40 (0.40)	0.56 (0.34)	–
Black	237/195	− 0.03 (0.31)	0.02 (0.29)	–
Project				
Project 1	445/0	0.20 (0.67)	–	–
Project 2	1059/1057	− 0.01 (0.58)	–	–
Birthyear				
Min		− 1.65 (1.11)	− 0.18 (0.77)	–
Max		0.07 (0.62)	6.35 (3.22)	–
Age				
(per month)		0.03 (0.02)	− 0.01 (0.02)	–

N is the number of animals within a group (presented as LE/(SLE and MC) since SLE and MC were only recorded in Project 2) and standard errors for the estimates are given between brackets. Two animals in project 2 were not phenotyped for SLE and MC. For the birth year effects (n = 11), the range of the estimates is shown. “–” indicates that the effect was not included in the estimated model

### Genome wide association study

In the case control approach, 241 cases (sensitive) and 192 controls (resilient) were identified following the previously described criteria. Average LE and SLE in cases were 15.15% (SD = 8.24%) and 9.88% (SD = 8.26%), respectively. For controls, average LE and SLE were 1.13% (SD = 1.20%) and 0.74% (SD = 0.99%), respectively.

**Fig. 2:**
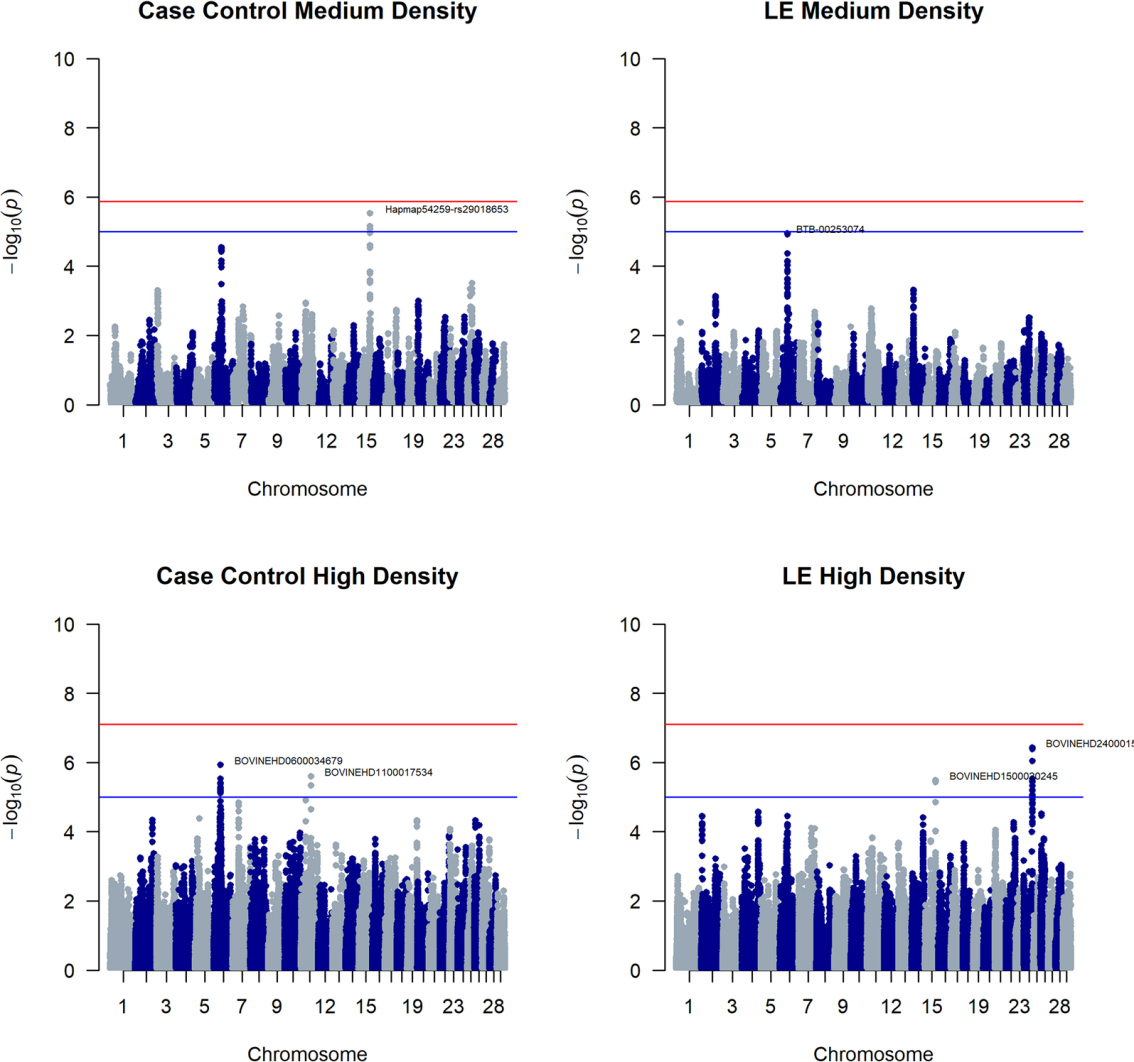
Manhattan plots of four haplotype-based GWAS for mange susceptibility in Belgian Blue. Top left shows the case–control approach on the medium density dataset (29,010 SNPs), top right shows the quantitative (lesion extent, LE) approach on medium density, bottom left the case–control approach on the high density dataset (633,512SNPs) and bottom right the quantitative analysis on high density

**Fig. 3:**
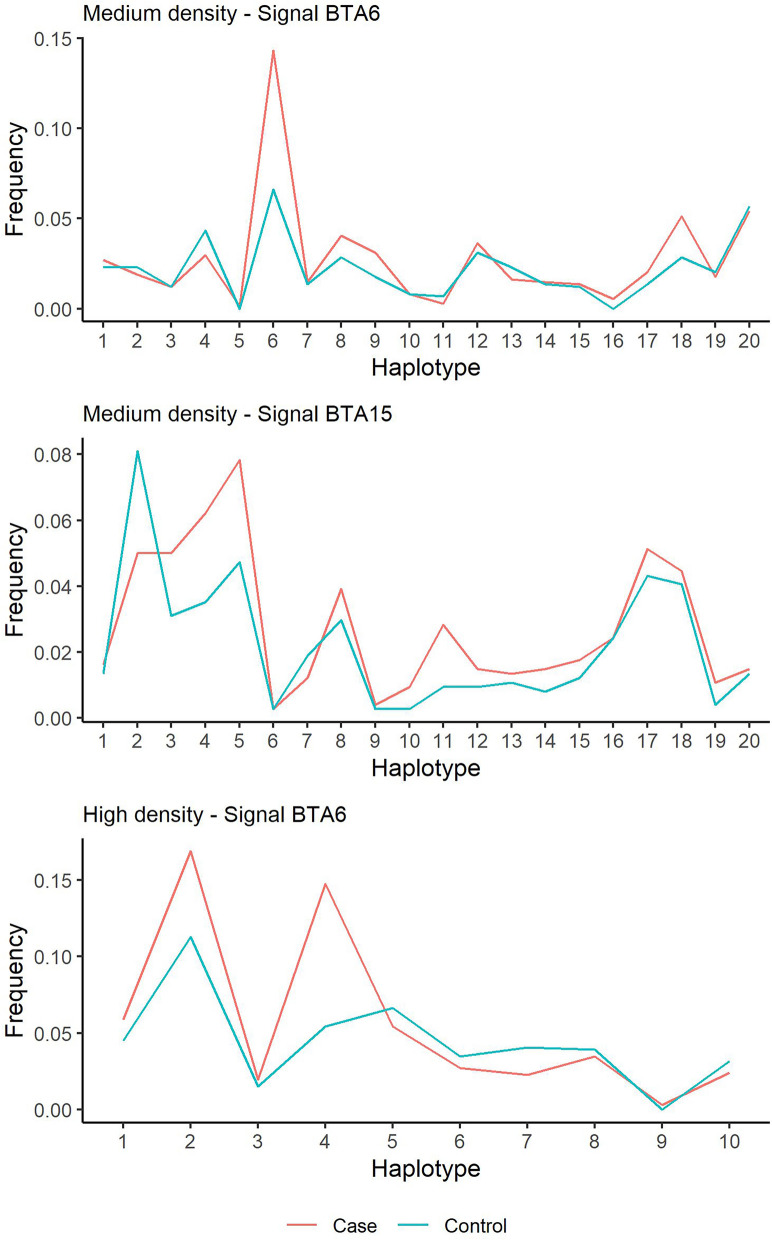
Frequency of inferred ancestral haplotypes within cases and controls for the top SNPs. (top) shows top SNP of the signal on BTA6, (middle) the top SNP of the signal on BTA15 in the medium density analysis, and (bottom) the top SNP on BTA6 in the high density analysis. Large differences between cases and controls could indicate an associated haplotype to psoroptic mange sensitivity at the top SNP position of the signal

Manhattan plots (Fig. 2) (QQ-plots are added in Additional file 5 Fig. S4) reveal for the case/control analysis at medium SNP density two signals: on BTA6 (pos: 43.7 Mbp) and BTA15 (pos: 67.8Mbp). The signal on BTA15 almost reaches chromosome-wide significance. Both signals were examined further, and haplotype frequencies within cases and controls at the most associated positions were compared (Fig. 3). For the BTA6 signal, one of the 20 inferred ancestral haplotypes had a frequency difference of 7.7% between cases and controls, suggesting the presence of a causal variant linked to this haplotype. For BTA15, three haplotypes had a difference of more than 3% between cases and controls. For the GWAS on LE, a signal on BTA6 (pos 43.7 Mbp) reached the suggestive threshold. GWAS results on SLE and MC are presented in Additional file 6 Fig. S5 but did not reveal any (suggestive) signals. Moreover, the analysis on mite count showed a minor discrepancy between expected and observed p-values (QQ plots in Fig. S6) indicating signs of stratification in the model. This could be explained by the distribution skewedness of the phenotypes (large number of zeros). However, generalized linear mixed models, such as our models used in GLASCOW, are generally robust against violations of non-normality can handle binary traits or counts (Poisson) and can often account for the non-independence of observations [27]. A log-transformation of LE, SLE and MC did not improve the outcome of the GWAS and those results were disregarded.

When imputed to high density SNP level (633,512 SNPs), the case/control analysis showed the same suggestive signal on BTA6 (pos: 42.9 Mbp). Also, two ancestral haplotypes had divergent frequencies between cases and controls (9.3% and 5.6% difference for haplotype 2 and 4 respectively). Moreover, a signal on BTA11 (pos: 61.9 Mbp) was detected. However, it has to be noted that the QQ-plot (Additional file 5 Fig. S4) detected some signs of stratification in this analysis that were not picked up by GLASCOW and might have originated from LD with the signal. The quantitative analysis on LE showed the same signal on BTA6, although this signal was not significant. However, the quantitative analysis showed a suggestive signal on both BTA15 (pos: 70.1 MBp) and BTA24 (54.6 MBp). To confirm the quantitative analyses on LE, haplotypes at the peak signals were fitted in an animal model (using remlf90) as levels of a fixed effect (Fig. 3). However, none of the haplotypes was found to significantly increase LE.

In summary, our analyses show four signals on BTA6, BTA11, BTA15 and BTA24. Underlying genes were identified and a complete list is given in Additional file 8 Tables S2 to S9. From this list, several suggestive candidate genes were detected based on their known function, namely: *GBA3* on BTA6, and *RAG2* and *TRAF6* on BTA 15.

## Discussion

This study is the first to examine the genetic background of the susceptibility of Belgian Blue cattle for psoroptic mange, a skin disease that bedevils this well performing beef breed. We evaluate genetic parameters for LE, SLE and MC, and detected candidate genes in the bovine genome associated to psoroptic mange susceptibility. These findings provide a first insight into the complex genetic background of psoroptic mange and indicate that breeding towards mange resilience is possible.

### Descriptive statistics

We succeeded to phenotype 1975 Belgian Blue cattle in more than 100 different management groups. Although we sampled a large number of animals, not all of them were included in the subsequent analyses. A total of 471 animals were unregistered in their respective herdbooks, resulting in unavailable birth dates. Consequently, recording their age at the time of sampling became impossible. Moreover, as SLE and MC were only scored in Project 2, genetic parameters for these traits could only be estimated on a subset of the sampled animals (n = 1057). We included only herds with clinical signs of psoroptic mange and observable differences in infestation level, as our goal was to detect individual differences between *P. ovis* infestation sensitivity between animals from the same environment. This resulted in an average LE of 5.85% and 3.38% SLE, with a sizeable proportion of animals with 0% LE or SLE (5.6% and 15.7% of all sampled animals, respectively). Therefore, the distributions of LE and SLE were skewed, similar to the study of Meyermans et al*.* in dual-purpose Belgian Blue cattle [15], although the mean lesion extent and SD are higher in the current study, which was expected as the dual-purpose population is considered being less susceptible to psoroptic mange.

Although anecdotal reports pointed towards a correlation between skinfold thickness and psoroptic mange susceptibility, we found that skinfold thickness was both phenotypically and genetically not correlated to (S)LE and MC, although the SEs of the genetic correlations were high. Previous research showed Belgian Blues have a relatively thin skin [28]. Therefore, a correlation between skin thickness and psoroptic mange susceptibility seemed plausible. Such a relationship would have been valuable, as skin thickness of breeding animals is routinely scored by technicians and a favorable correlation to LE would allow an easy incorporation of mange sensitivity in the Belgian Blue breeding program.

33% of the animals were sampled twice with a two-week interval. Correlations for LE and SLE between two successive visits were high, indicating that our measurements are repeatable. This repeatability is a good indication that LE and SLE are useful phenotypes for routine screening of psoroptic mange susceptibility. Not all animals could be screened for a second time (n = 665), as some farmers did not allow postponing the acaricide treatment after the first visit.

### Genetic parameters for psoroptic mange

The estimated h^2^ for LE in Belgian Blue cattle was low, but higher for SLE. Therefore SLE could be used in a breeding program against psoroptic mange susceptibility as it is also easier to score, compared to mild lesions (class 1 and 2, see Additional file 2 Fig. S1). Thus, a large scale phenotyping protocol could focus on recording animals with severe, wet and crusty lesions. Table 2 shows a clear role for the CG effect on LE and SLE (c_g_^2^ = 0.420 and 0.494, respectively), and to a lesser extent on MC (c_g_^2^ = 0.149), showing that management still plays an important role in psoroptic mange susceptibility. In this model we estimated CG as a random effect, to allow the estimation of c_g_^2^ for each of the traits. We also assessed the same model with CG as a fixed effect. This led to slightly higher h^2^ estimates: 0.072 for LE, 0.13 for SLE, and 0.16 for MC. This outcome was expected, as CG may account for a small portion of the additive genetic variance. Estimates for individual CG were highly correlated (r = 0.87–0.93) between these two models. Despite these differences, the heritability estimates from both models did not substantially differ. Consequently, we chose to present the model with CG as a random effect, allowing us to estimate c_g_^2^. It is evident that CG plays a pivotal role in the development of psoroptic mange, including factors such as management conditions and age within a CG. However, for MC, this role appears to be more limited (c_g_^2^ = 0.149).

As psoroptic mange lesions evolve over time, the timing of sampling is a crucial variable. We captured this variability as much as possible by performing the analysis on the CG level, under the assumption that each animal within the CG had a similar exposure to *P. ovis*. Even within the variability of the moment of observing (S)LE and MC within a CG, we were still able to capture a significant portion of heritability of psoroptic mange susceptibility. With our analysis we demonstrate that, irrespective of the precise stage of the infestation and lesion development, our methodology was successful in identifying a substantial part of the genetic variation in lesion development and susceptibility to psoroptic mange in Belgian Blue cattle.

In literature, most studies report low h^2^ for endo- and ectoparasitic susceptibility in cattle [10, 12, 13]. Only Burrow found moderate to high h^2^ for tick and gastrointestinal nematode load in tropical beef cattle [11]. However, all animals in Burrow’s study originated from the same research station, which could partly explain the low environmental variance estimate. This was also observed by Meyermans and colleagues [15] in the dual purpose Belgian Blue population, where estimated h^2^ was higher (12.7% in the dual-purpose population) and CG variances were lower. This lower environmental variance was expected, as the dual-purpose herds sampled in [15] were housed inside (dairy-type farms) with more environmental control. In the current study, all sampled cattle were managed in a free range system in spring, summer and early autumn, where environmental effects can be larger. Moreover, May et al*.* found similarly large environmental effects in analyzing endoparasite infections in German dairy cattle [12].

Genetic correlations between LE and SLE were moderate (0.40), indicating that LE and SLE possibly represent different aspects of psoroptic mange susceptibility. Likewise, genetic correlations between MC and (S)LE were low, and therefore also these phenotypes appear to be partial different aspects of psoroptic mange sensitivity. This low association hence suggests that the number of mites is not necessarily linked to the severity of the infection. The estimated correlation per CG between LE and SLE was high (0.90), showing that environments with high incidences of LE also have high incidences of SLE. CG correlations for (S)LE and MC were moderately high, showing that the environmental effect on *P. ovis* presence and the development of lesions (e.g. treatment management, housing conditions) vary mostly in the same direction but are not identical.

In the animal model, fixed effects estimates are presented in Table 3. For these estimates, it has to be noted that with the exception of the sex effects, SEs were relatively high. Males had larger lesions and higher mite counts than females, although it has to be noted that we sampled more females than males. In practice, there are few indications that males have a higher predisposition to psoroptic mange. Coat color had a small effect on (S)LE where white animals had slightly larger lesions than black and blue coated animals, and blue coats had also larger (S)LE than black coats. The same trend was previously observed in the dual purpose Belgian Blue population [15].

### Genome wide association study

In this study, different GWAS were performed based on either a case/control or quantitative approach (LE, SLE and MC) on either medium density or imputed high density genotypes. For selecting cases and controls, we chose to directly identify extremes at the CG level, without extensive pre-adjustments. This decision was influenced by the limited availability of correction factors, the relatively small sizes of some CGs, and was taken to correct for a potential variation in the moment of sampling as discussed earlier.

Some of these analyses pointed to identical signals (e.g. BTA6 signal was detected in all analyses) whereas others were not corroborated in different analyses (e.g. BTA24 signal only found in LE high density analysis). Of the detected signals, none reached Bonferroni-corrected, genome wide significance. However, for the BTA6 and BTA15 signals detected in both case–control analyses, haplotypes were identified with an increased prevalence in cases. Detection of these haplotypes suggests the presence of causative mutations in the underlying regions. The fact that the estimates fitted in the animal model did not show a clear haplotype associated to LE, is in line with the fact that the discovered signals were not always genome-wide significant. Overall, we conclude that psoroptic mange susceptibility is largely under polygenic control, as no single major QTL was detected throughout the analysis. On BTA11 and BTA24, no obvious candidate genes were identified during the literature review. However for BTA6 and BTA15, some promising candidate genes were detected.

The first candidate gene is *GBA3* (Glucosylceramidase Beta 3), located on BTA6. *GBA3* plays a role in the glucosylceramide metabolism and deficiency of *GBA3* is, in humans, causal for Gaucher disease [29]. This deficiency results in affected macrophages due to accumulation of glucocerebrosides. It causes increased susceptibility to various infestations, enlarged liver and spleen, and an impaired skin barrier system [30]. Glucocerebrosidases play an important role in the integrity of the epidermis and disruption in a mouse-model caused increased epidermal water loss and resulted in an increase flux of exogenous compounds through the skin [31]. Therefore, *GBA3* has been linked to atopic dermatitis and Netherton syndrome (scaling skin, hair abnormalities and increased susceptibility to atopic dermatitis) [32, 33]. Based on this literature and its link to immunology and skin barrier, *GBA3* is a good candidate gene for further investigation.

Two very strong candidate genes are underlying the signal on BTA15: *RAG2* (Recombination activating gene 2 protein) and *TRAF6* (TNF receptor associated factor 6). Together with the adjacent *RAG1* gene, *RAG2* plays a crucial role in the V(D)J recombination which is crucial in the early T- and B-cell maturation and is therefore vital for the generation of antibody diversity [34]. In humans, *RAG2* deficiencies are linked to the Omenn syndrome [35], a severe immunodeficiency with clinical symptoms such as erythematous rash, hepatosplenomegaly, desquamation, alopecia and susceptibility to recurrent infections [36]. In mice, disruption of the *RAG* complex results in the suspension of T- and B-cell development [37]. Moreover, mutations in *RAG* genes have been associated to severe combined immunodeficiency with e.g. skin granulomas [38]. As this gene plays a vital role in the immune system, especially in skin and dermis, it is a clear candidate gene for further analysis in psoroptic mange susceptibility.

Near the *RAG2* locus on BTA15, a third candidate gene *TRAF6* is located. *TRAF6* is a gene that plays a central role in the signal transduction of toll-like receptors, a crucial part of the innate immune system. *TRAF6* is part of the TNF-receptor superfamily and is a signal transducer in the NFκB pathway, reacting to pro-inflammatory cytokines. Therefore, it is crucial for the development, homeostasis and activation of T- and B-cells, macrophages, dendritic cells and other myeloid cells [39].

This study provides the identification of three strong candidate genes for psoroptic mange susceptibility in Belgian Blue cattle, but since not all analyses found the same signals and not all signals were conclusive, future developments in this research should go two-fold. First, the discovered signals can be evaluated at whole-genome sequence resolution. For example, for the signal found on BTA6, two haplotypes were more common in cases. It could be checked whether these two haplotypes share a common mutation that is not present in animals that were identified as controls. Second, the inclusion of more animals via routine evaluation of psoroptic mange susceptibility of cattle could lead to the identification of new cases and controls and a more accurate estimate of the genetic parameters. While we successfully phenotyped and sampled a substantial number of animals (n = 1975), not all animals could be included in every analysis. As our dataset contained incomplete records or animals that were not screened for all traits, genetic parameters were only estimated for a subset of these 1975 cattle. Therefore, a routine screening following our screening protocol could resolve such issues and would allow the development of breeding values against psoroptic mange susceptibility. Moreover, as genotyping breeding animals is more common these days, such information could be added to the analysis, to provide genomic breeding values and further investigate the identified signals in this study.

## Conclusions

Sensitivity for psoroptic mange in Belgian Blue cattle is an important economic and animal welfare issue. Based on a large cohort of animals, we were able to estimate the heritability of psoroptic mange, quantified as lesion extent, severe lesion extent and/or mite count. Heritability for mange lesion extent and for severe mange lesions was low, whereas heritability was moderate for psoroptic mite count. A haplotype based GWAS using both a case–control and quantitative approach revealed several suggestive signals and *GBA3*, *RAG2* and *TRAF6* were identified as candidate genes for further examination. These genes are linked to atopic dermatitis and play a crucial role in the immune system, and therefore this skin disease in Belgian Blue cattle could serve as model for other species (including humans) to (ectoparasitic) skin diseases. However, our results indicate psoroptic mange sensitivity is under polygenic control, as not one single major gene could be detected, but instead it is more probable that a large number of genes have a small effect on psoroptic mange. Further, sequence based studies could reveal the causal mutations associated with the detected haplotypes. This research is the first step towards the genomic characterization of psoroptic mange susceptibility and towards breeding for less susceptible animals.

### Supplementary Information

Additional file 1: Figure S1. Four types of psoroptic mange lesions in Belgian Blue cattle, ranging from score 1: healing/healed lesions, to score 4: the most severe lesions with wound exudate and crusts.

Additional file 2: Table S1. Detailed descriptive statistics per lesion score group for all phenotyped Belgian Blue cattle.

Additional file 3: Figure S2. Histograms of the four different lesion scores of 1306 phenotyped Belgian Blue cattle. These detailed lesion scores were only given in Project 2 and lesion sizes are expressed as percentage of body coverage.

Additional file 4: Figure S3. Histogram of mite count for Belgian Blue cattle phenotyped in project 2 (n = 1306) in a skin scraping of 12 cm^2^ at the predilection sites for *P. ovis*. For one animal, mite count was topped at 1000 live *P. ovis* mites.

Additional file 5: Figure S4. QQ-plots of the four haplotype-based GWAS for mange susceptibility in Belgian Blue. Top left shows the case–control approach on the medium density dataset (29,010 SNPs), top right shows the quantitative (lesion extent, LE) approach on medium density, bottom left the case–control approach on the high density dataset (633,512 SNPs) and bottom right the quantitative analysis on high density.

Additional file 6: Figure S5. Manhattan plots for severe lesion extent (A) and mite count (B) on the medium density dataset (29,010 SNPs).

Additional file 7: Figure S6. QQ-plots for severe lesion extent (A) and mite count (B) on the medium density dataset (29,010 SNPs).

Additional file 8: Table S2. Overview of genes in the 4Mbp range of BTA6’s top SNP in the case control on the medium density analysis: ARS-BFGL-NGS-58762 on position 43,713,187. Table S3. Overview of genes in the 4Mbp range of BTA15’s top SNP in the case control on the medium density analysis: Hapmap54259-rs29018653 on position 67,816,375. Table S4. Overview of genes in the 4Mbp range of BTA6’s top SNP in the quantitative analysis on Lesion Extent on the medium density data: BTB-00253074 on position 43,811,863. Table S5. Overview of genes in the 4Mbp range of BTA6’s top SNP in the case control analysis on high density imputed data: BOVINEHD0600034679 on position 42,861,985. Table S6. Overview of genes in the 4Mbp range of BTA11’s top SNP in the case control analysis on high density imputed data: BOVINEHD1100017534 on position 61,865,790. Table S7. Overview of genes in the 4Mbp range of BTA6’s top SNP in the quantitative analysis on Lesion Extent on high density imputed data: BOVINEHD0600013002 on position 47,540,677. Table S8. Overview of genes in the 4Mbp range of BTA15’s top SNP in the quantitative analysis on Lesion Extent on high density imputed data: BOVINEHD1500020245 on position 70,129,820. Table S9. Overview of genes in the 4Mbp range of BTA24’s top SNP in the quantitative analysis on Lesion Extent on high density imputed data: BOVINEHD2400015598 on position 54,644,915

## Data Availability

The generated data are partially owned by the respective herdbook associations and restrictions may apply to the availability of the data. Motivated requests for data to the corresponding author will be forwarded to the respective herdbooks.
