# Supplementary material for: Genetic and genomic analysis of Belgian Blue’s susceptibility for psoroptic mange
Source: Genet Sel Evol. 2024 Jul 5;56:52. doi: 10.1186/s12711-024-00921-7 (PMC11227209; doi:10.1186/s12711-024-00921-7)
Supplement: Supplementary file 1 — Additional file 1: Figure S1. Four types of psoroptic mange lesions in Belgian Blue cattle, ranging from score 1: healing/healed lesions, to score 4: the most severe lesions with wound exudate and crusts. [file 12711_2024_921_MOESM1_ESM.pdf]

Additional file 1 Figure S1

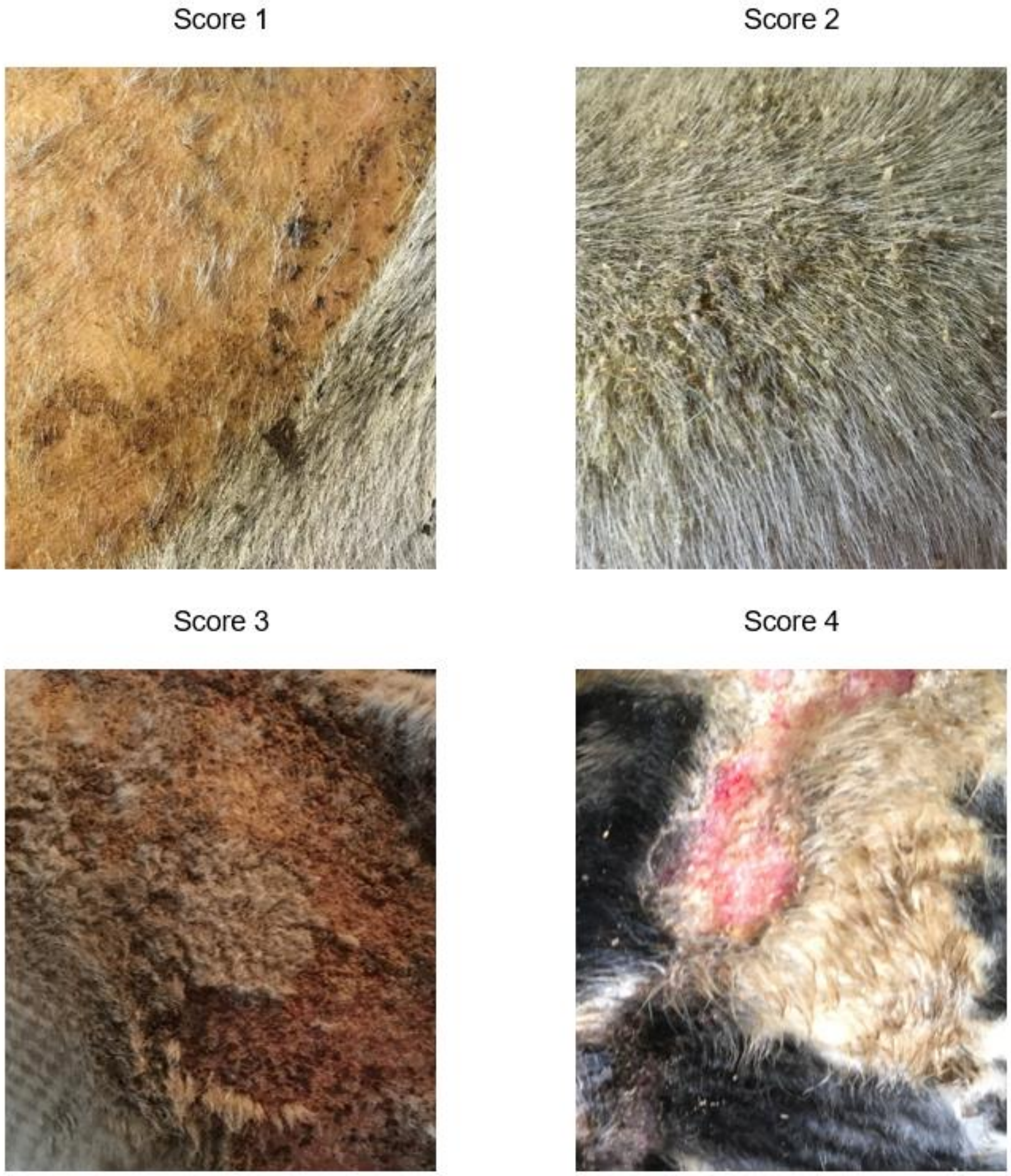

Four types of psoroptic mange lesions in Belgian Blue cattle, ranging from score 1: healing/healed lesions, to score 4: the most severe lesions with wound exudate and crusts.
