# Supplementary material for: Genetic and genomic analysis of Belgian Blue’s susceptibility for psoroptic mange
Source: Genet Sel Evol. 2024 Jul 5;56:52. doi: 10.1186/s12711-024-00921-7 (PMC11227209; doi:10.1186/s12711-024-00921-7)
Supplement: Supplementary file 2 — Additional file 2: Table S1. Detailed descriptive statistics per lesion score group for all phenotyped Belgian Blue cattle. [file 12711_2024_921_MOESM2_ESM.pdf]

Additional file 2 Table S1

Detailed descriptive statistics per lesion score group for all phenotyped Belgian Blue cattle. Total lesion extent is the summed lesion extent of all different scores and severe lesion extent the combined lesion extent of score 3 and 4. Detailed lesion scores were only given in Project 2 (n=1306). Lesion extent is expressed as percentage of body coverage.

	Mean	Median	Range	Variance
Lesion Extent (in %)	5.85	3.72	0 – 53.14	41.42
Severe Lesion Extent (in %)	3.38	1.43	0 – 53.14	26.57
Lesion extent 1	0.48	0	0 – 25.14	4.14
Lesion extent 2	1.89	0.86	0 – 38.00	8.08
Lesion extent 3	2.17	0.86	0 – 24.86	10.38
Lesion extent 4	1.22	0	0 – 48.00	14.95
