# Supplementary material for: Genetic and genomic analysis of Belgian Blue’s susceptibility for psoroptic mange
Source: Genet Sel Evol. 2024 Jul 5;56:52. doi: 10.1186/s12711-024-00921-7 (PMC11227209; doi:10.1186/s12711-024-00921-7)
Supplement: Supplementary file 3 — Additional file 3: Figure S2. Histograms of the four different lesion scores of 1306 phenotyped Belgian Blue cattle. These detailed lesion scores were only given in Project 2 and lesion sizes are expressed as percentage of body coverage. [file 12711_2024_921_MOESM3_ESM.pdf]

Additional file 3 Figure S2

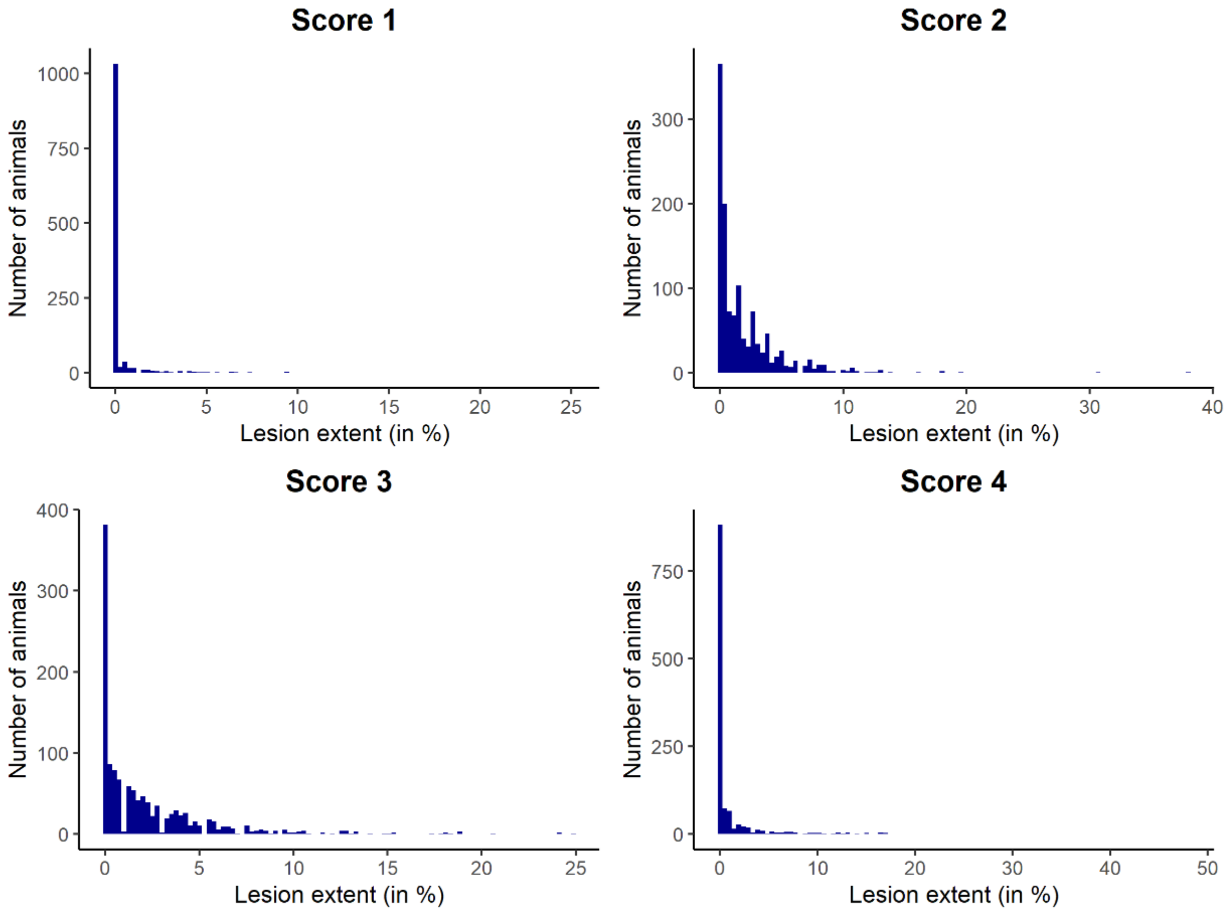

Histograms of the four different lesion scores of 1306 phenotyped Belgian Blue cattle. These detailed lesion scores were only given in Project 2 and lesion extent is expressed as percentage of body coverage.
