# Supplementary material for: Genetic and genomic analysis of Belgian Blue’s susceptibility for psoroptic mange
Source: Genet Sel Evol. 2024 Jul 5;56:52. doi: 10.1186/s12711-024-00921-7 (PMC11227209; doi:10.1186/s12711-024-00921-7)
Supplement: Supplementary file 5 — Additional file 5: Figure S4. QQ-plots of the four haplotype-based GWAS for mange susceptibility in Belgian Blue. Top left shows the case–control approach on the medium density dataset (29,010 SNPs), top right shows the quantitative (lesion extent, LE) approach on medium density, bottom left the case–control approach on the high density dataset (633,512 SNPs) and bottom right the quantitative analysis on high density. [file 12711_2024_921_MOESM5_ESM.pdf]

Additional file 5 Figure S4

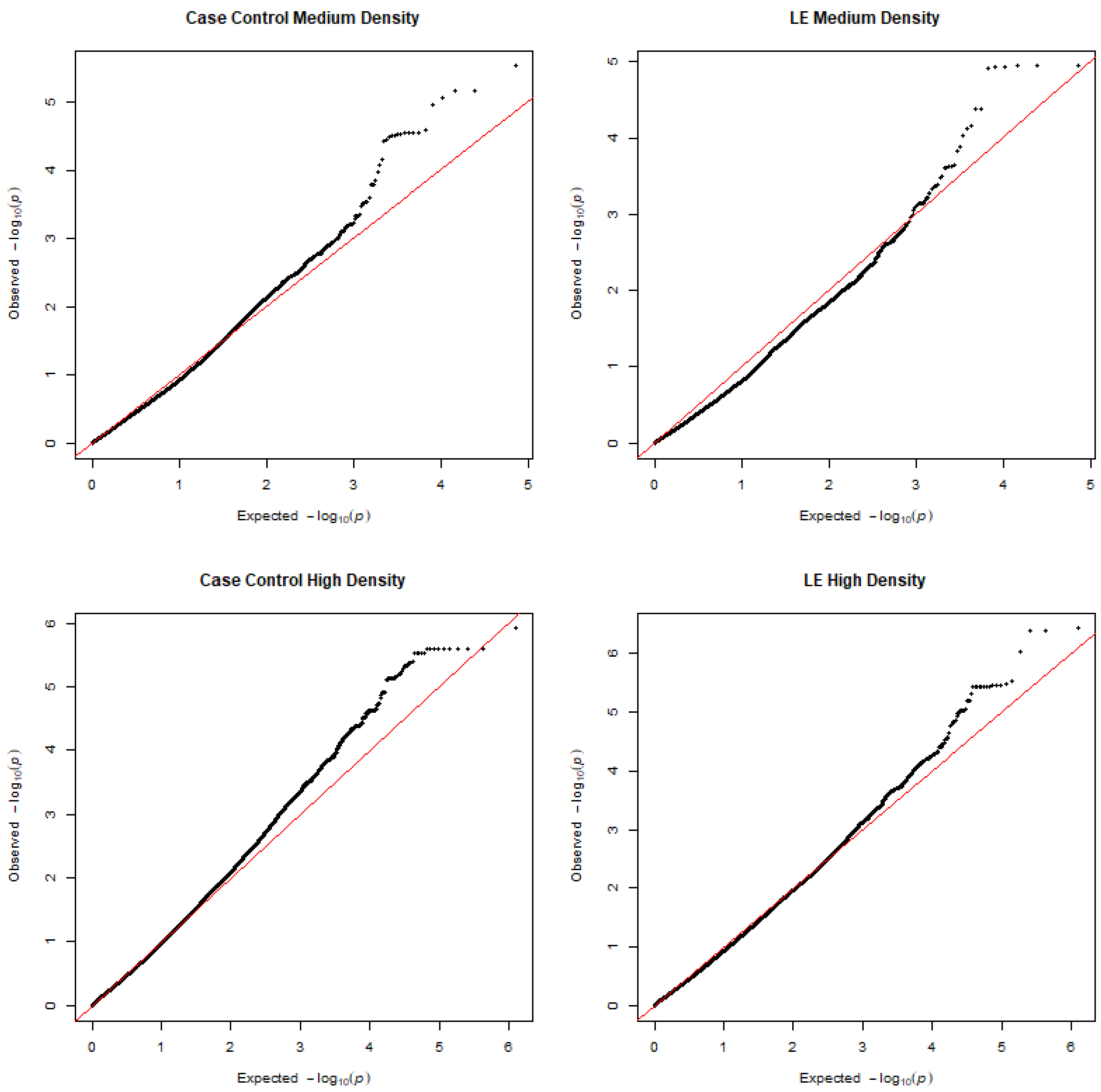

QQ-plots of the four haplotype-based GWAS for mange susceptibility in Belgian Blue. Top left shows the case-control approach on the medium density dataset (29,010 SNPs), top right shows the quantitative (lesion extent, LE) approach on medium density, bottom left the case-control approach on the high density dataset (633,512 SNPs) and bottom right the quantitative analysis on high density.
