# Supplementary material for: Genetic and genomic analysis of Belgian Blue’s susceptibility for psoroptic mange
Source: Genet Sel Evol. 2024 Jul 5;56:52. doi: 10.1186/s12711-024-00921-7 (PMC11227209; doi:10.1186/s12711-024-00921-7)
Supplement: Supplementary file 7 — Additional file 7: Figure S6. QQ-plots for severe lesion extent (A) and mite count (B) on the medium density dataset (29,010 SNPs). [file 12711_2024_921_MOESM7_ESM.pdf]

Additional file 7 Figure S6

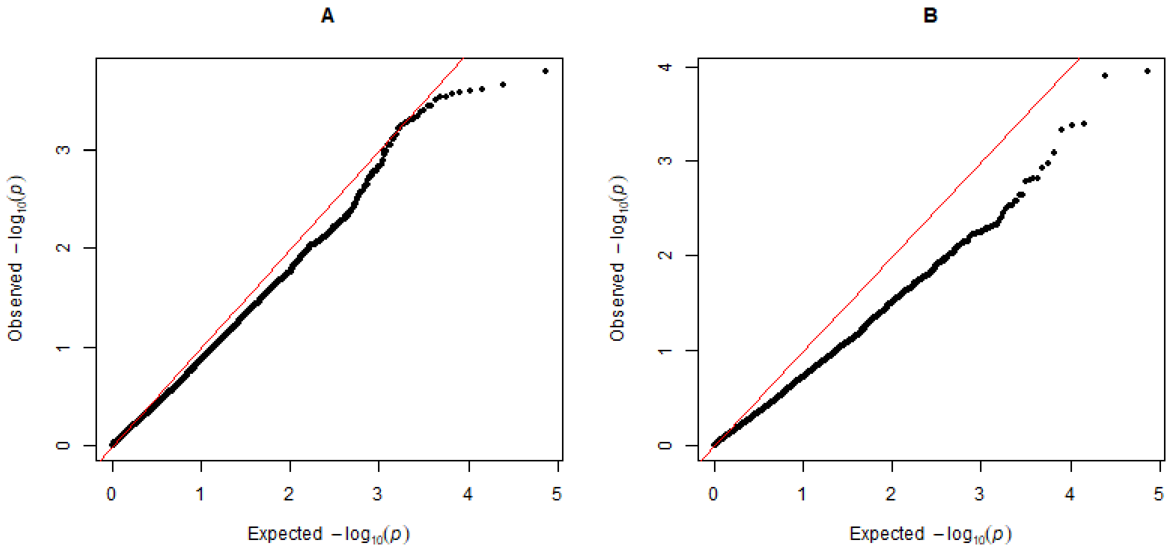

QQ-plots for severe lesion extent (A) and mite count (B) on the medium density dataset (29,010 SNPs).
