# Supplementary material for: Genetic and genomic analysis of Belgian Blue’s susceptibility for psoroptic mange
Source: Genet Sel Evol. 2024 Jul 5;56:52. doi: 10.1186/s12711-024-00921-7 (PMC11227209; doi:10.1186/s12711-024-00921-7)
Supplement: Supplementary file 8 — Additional file 8: Table S2. Overview of genes in the 4Mbp range of BTA6’s top SNP in the case control on the medium density analysis: ARS-BFGL-NGS-58762 on position 43,713,187. Table S3. Overview of genes in the 4Mbp range of BTA15’s top SNP in the case control on the medium density analysis: Hapmap54259-rs29018653 on position 67,816,375. Table S4. Overview of genes in the 4Mbp range of BTA6’s top SNP in the quantitative analysis on Lesion Extent on the medium density data: BTB-00253074 on position 43,811,863. Table S5. Overview of genes in the 4Mbp range of BTA6’s top SNP in the case control analysis on high density imputed data: BOVINEHD0600034679 on position 42,861,985. Table S6. Overview of genes in the 4Mbp range of BTA11’s top SNP in the case control analysis on high density imputed data: BOVINEHD1100017534 on position 61,865,790. Table S7. Overview of genes in the 4Mbp range of BTA6’s top SNP in the quantitative analysis on Lesion Extent on high density imputed data: BOVINEHD0600013002 on position 47,540,677. Table S8. Overview of genes in the 4Mbp range of BTA15’s top SNP in the quantitative analysis on Lesion Extent on high density imputed data: BOVINEHD1500020245 on position 70,129,820. Table S9. Overview of genes in the 4Mbp range of BTA24’s top SNP in the quantitative analysis on Lesion Extent on high density imputed data: BOVINEHD2400015598 on position 54,644,915 [file 12711_2024_921_MOESM8_ESM.pdf]

Additional file 8

Table S2: Overview of genes in the 4Mbp range of BTA6's top SNP in the case control on the medium density analysis: ARS-BFGL-NGS-58762 on position 43,713,187

**Ensembl gene ID**	**Name**	**Start position**	**End position**
ENSBTAG00000042427	*RF00026*	42,431,795	42,431,898
ENSBTAG00000004653	*ADGRA3*	43,436,544	43,531,420
ENSBTAG00000020363	*GBA3*	43,675,600	43,863,550
ENSBTAG00000042453	*RF00026*	44,553,810	44,553,916
ENSBTAG00000033327		44,668,706	44,669,207
ENSBTAG00000017024	*PPARGC1A*	44,854,113	44,960,668
ENSBTAG00000043366	*RF00026*	45,527,603	45,527,709
ENSBTAG00000008896	*DHX15*	45,629,617	45,683,587

Table S3: Overview of genes in the 4Mbp range of BTA15's top SNP in the case control on the medium density analysis: Hapmap54259-rs29018653 on position 67,816,375

**Ensembl gene ID**	**Name**	**Start position**	**End position**
ENSBTAG00000012460	*ELF5*	65,824,442	65,854,386
ENSBTAG00000017150	*EHF*	65,991,132	66,030,964
ENSBTAG00000018257	*APIP*	66,207,587	66,231,698
ENSBTAG00000018261	*PDHX*	66,231,837	66,307,403
ENSBTAG00000044322	*bta-mir-1343*	66,264,848	66,264,925
ENSBTAG00000011578	*CD44*	66,454,331	66,541,790
ENSBTAG00000012628	*SLC1A2*	66,580,653	66,637,083
ENSBTAG00000012630	*PAMR1*	66,754,734	66,832,246
ENSBTAG00000047083	*FJX1*	66,929,952	66,930,443
ENSBTAG00000037389	*TRIM44*	66,987,987	67,099,792
ENSBTAG00000044158	*LDLRAD3*	67,240,027	67,518,041
ENSBTAG00000019045	*COMMD9*	67,563,703	67,576,723
ENSBTAG00000021029	*PRR5L*	67,661,945	67,741,048
ENSBTAG00000036009	*TRAF6*	67,768,374	67,789,469
ENSBTAG00000040293	*RAG1*	67,827,233	67,830,364
ENSBTAG00000031309	*RAG2*	67,841,773	67,849,540
ENSBTAG00000021611	*C15H11orf74*	67,844,540	67,911,151
ENSBTAG00000044849	*RF00026*	67,874,695	67,874,766
ENSBTAG00000001623		68,996,828	69,000,079

Table S4: Overview of genes in the 4Mbp range of BTA6’s top SNP in the quantitative analysis on Lesion Extent on the medium density data: BTB−00253074 on position 43,811,863

**Ensembl gene ID**	**Name**	**Start position**	**End position**
ENSBTAG00000042427	*RF00026*	42,431,795	42,431,898
ENSBTAG00000004653	*ADGRA3*	43,436,544	43,531,420
ENSBTAG00000020363	*GBA3*	43,675,600	43,863,550
ENSBTAG00000042453	*RF00026*	44,553,810	44,553,916
ENSBTAG00000033327		44,668,706	44,669,207
ENSBTAG00000017024	*PPARGC1A*	44,854,113	44,960,668
ENSBTAG00000043366	*RF00026*	45,527,603	45,527,709
ENSBTAG00000008896	*DHX15*	45,629,617	45,683,587

Table S5: Overview of genes in the 4Mbp range of BTA6's top SNP in the case control analysis on high density imputed data: BOVINEHD0600034679 on position 42,861,985

Ensembl gene ID	Name	Start position	End position
ENSBTAG00000005108	SLIT2	41,236,589	41,640,789
ENSBTAG00000029783	bta-mir-218-1	41,545,029	41,545,138
ENSBTAG00000033351	PACRGL	41,685,691	41,707,024
ENSBTAG00000047743	KCNIP4	41,709,362	43,021,626
ENSBTAG00000042427	RF00026	42,431,795	42,431,898
ENSBTAG00000004653	ADGRA3	43,436,544	43,531,420
ENSBTAG00000020363	GBA3	43,675,600	43,863,550
ENSBTAG00000042453	RF00026	44,553,810	44,553,916
ENSBTAG00000033327		44,668,706	44,669,207

Table S6: Overview of genes in the 4Mbp range of BTA11's top SNP in the case control analysis on high density imputed data: BOVINEHD1100017534 on position 61,865,790

Ensembl gene ID	Name	Start position	End position
ENSBTAG00000013254	*XPO1*	60,068,221	60,109,922
ENSBTAG00000039218		60,268,166	60,268,762
ENSBTAG00000009290	*FAM161A*	60,352,576	60,381,305
ENSBTAG00000004531	*CCT4*	60,397,422	60,410,035
ENSBTAG00000019867	*COMMD1*	60,427,663	60,598,437
ENSBTAG00000018046	*B3GNT2*	60,654,634	60,687,730
ENSBTAG00000000899	*TMEM17*	60,934,735	60,942,371
ENSBTAG00000044173	*EHBP1*	61,117,376	61,472,058
ENSBTAG00000006760	*OTX1*	61,479,110	61,482,379
ENSBTAG00000023318		61,716,757	61,717,260
ENSBTAG00000005151	*WDPCP*	61,759,950	61,969,748
ENSBTAG00000019295	*MDH1*	61,970,467	61,994,479
ENSBTAG00000000111	*UGP2*	62,244,354	62,299,938
ENSBTAG00000000115	*VPS54*	62,301,309	62,377,281
ENSBTAG00000020865	*PELI1*	62,471,609	62,528,075
ENSBTAG00000000156	*LGALSL*	62,812,719	62,817,829
ENSBTAG00000009299	*AFTPH*	62,911,414	62,951,512
ENSBTAG00000007793	*SERTAD2*	62,992,908	62,993,855
ENSBTAG00000046117		63,290,422	63,395,507
ENSBTAG00000007763	*SLC1A4*	63,337,655	63,367,589
ENSBTAG00000016717	*CEP68*	63,413,128	63,424,655
ENSBTAG00000016720	*RAB1A*	63,429,725	63,458,565
ENSBTAG00000009761	*ACTR2*	63,552,997	63,591,980
ENSBTAG00000019456	*SPRED2*	63,639,742	63,672,178

Table S7: Overview of genes in the 4Mbp range of BTA6's top SNP in the quantitative analysis on Lesion Extent on high density imputed data: BOVINEHD0600013002 on position 47,540,677

Ensembl gene ID	Name	Start position	End position
ENSBTAG00000008896	*DHX15*	45,629,617	45,683,587
ENSBTAG00000043744	*RF00001*	45,845,700	45,845,802
ENSBTAG00000013980	*SOD3*	45,927,554	45,930,819
ENSBTAG00000021940	*CCDC149*	45,940,147	46,057,441
ENSBTAG00000017390	*LGI2*	46,127,372	46,155,777
ENSBTAG00000014616	*SEPSECS*	46,251,864	46,286,668
ENSBTAG00000006492	*PI4K2B*	46,355,300	46,391,439
ENSBTAG00000007240	*ZCCHC4*	46,415,046	46,464,861
ENSBTAG00000000646	*ANAPC4*	46,471,813	46,504,926
ENSBTAG00000028675	*RF00001*	46,501,972	46,502,082
ENSBTAG00000042665	*RF00026*	46,721,279	46,721,385
ENSBTAG00000001545	*SLC34A2*	46,724,553	46,749,777
ENSBTAG00000002299	*SEL1L3*	46,791,165	46,881,531
ENSBTAG00000003897	*SMIM20*	46,975,914	46,988,666
ENSBTAG00000045268		47,184,385	47,184,455
ENSBTAG00000003602	*RBPJ*	47,318,340	47,429,147
ENSBTAG00000043333	*RF00026*	47,351,713	47,351,816
ENSBTAG00000004988	*CCKAR*	47,468,324	47,479,538
ENSBTAG00000033214	*TBC1D19*	47,570,062	47,723,788
ENSBTAG00000019353	*STIM2*	47,941,559	48,006,406
ENSBTAG00000047735		48,036,646	48,036,732
ENSBTAG00000018885		48,314,930	48,315,299
ENSBTAG00000045570		48,464,280	48,464,799

Table S8: Overview of genes in the 4Mbp range of BTA15's top SNP in the quantitative analysis on Lesion Extent on high density imputed data: BOVINEHD1500020245 on position 70,129,820

Ensembl gene ID	Name	Start position	End position
ENSBTAG00000001623		68,996,828	69,000,079
ENSBTAG00000042407	*RF00026*	69,877,187	69,877,293
ENSBTAG00000029461	*RF00001*	71,047,556	71,047,665
ENSBTAG00000007324	*LRRC4C*	71,243,845	71,245,767

Table S9: Overview of genes in the 4Mbp range of BTA24's top SNP in the quantitative analysis on Lesion Extent on high density imputed data: BOVINEHD2400015598 on position 54,644,915

**Ensembl gene ID**	**Name**	**Start position**	**End position**
ENSBTAG00000037457	*MBD2*	54,044,363	54,093,578
ENSBTAG00000009129	*POLI*	54,154,068	54,178,071
ENSBTAG00000008168	*STARD6*	54,186,472	54,203,204
ENSBTAG00000008169	*C24H18orf54*	54,209,587	54,228,633
ENSBTAG00000046901		54,256,859	54,258,626
ENSBTAG00000042220	*RF00026*	54,290,445	54,290,551
ENSBTAG00000013871		54,470,149	54,474,339
ENSBTAG00000002788	*RAB27B*	54,544,871	54,715,264
ENSBTAG00000008158	*CCDC68*	54,728,527	54,786,445
ENSBTAG00000016462	*TCF4*	55,056,433	55,435,276
ENSBTAG00000045116	*RF00004*	55,115,179	55,115,351
ENSBTAG00000015731	*TXNL1*	56,404,067	56,446,678
