# Supplementary material for: Genetic and genomic analysis of Belgian Blue’s susceptibility for psoroptic mange
Source: Genet Sel Evol. 2024 Jul 5;56:52. doi: 10.1186/s12711-024-00921-7 (PMC11227209; doi:10.1186/s12711-024-00921-7)
Supplement: Supplementary file 4 — Additional file 4: Figure S3. Histogram of mite count for Belgian Blue cattle phenotyped in project 2 (n = 1306) in a skin scraping of 12 cm2 at the predilection sites for P. ovis. For one animal, mite count was topped at 1000 live P. ovis mites. [file 12711_2024_921_MOESM4_ESM.pdf]

Additional file 4 Figure S3

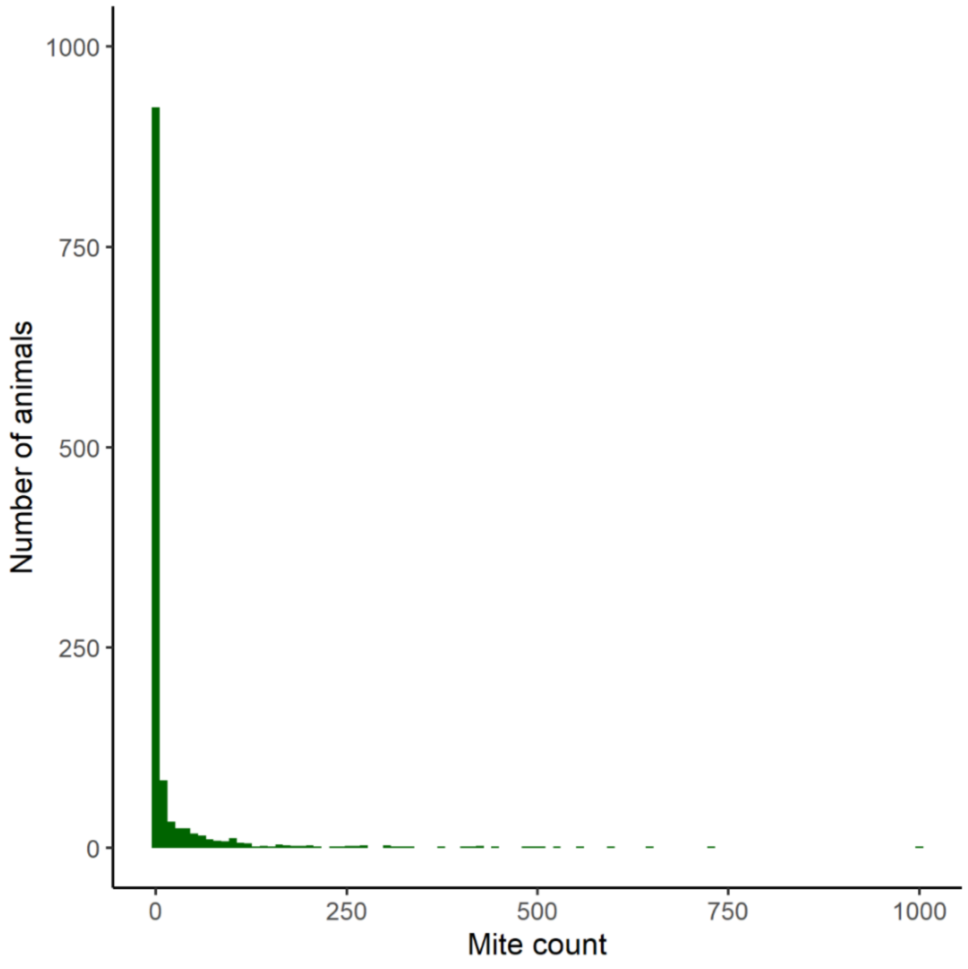

Histogram of mite count for Belgian Blue cattle phenotyped in project 2 (n= 1306) in a skin scraping of 12 cm² at the predilection sites for *P. ovis*. For one animal, mite count was topped at 1000 live *P. ovis* mites.
